# START: A Satellite Three Axis Rotation Testbed

**DOI:** 10.3390/mi13020165

**Published:** 2022-01-22

**Authors:** Giovanni Lavezzi, Nathan J. Stang, Marco Ciarcià

**Affiliations:** Department of Mechanical Engineering, South Dakota State University, Brookings, SD 57006, USA; nathan.stang@jacks.sdstate.edu (N.J.S.); marco.ciarcia@sdstate.edu (M.C.)

**Keywords:** satellite, attitude, simulator, testbed

## Abstract

The main goal of this paper is to illustrate the development of a satellite attitude simulator testbed for on-ground experimentation of attitude, determination, and control methodologies. This setup aims to be a low-cost alternative to testbeds based on air-bearing couplings. Our system is mainly composed of a suspended base, a single-board processor, a set of reaction wheels, and a battery. The suspension system entails a set of thin high-tensile strength wires converging on a single wire, which is in turn connected to the base. This configuration allows a three degrees-of-freedom rotation range and minimal resistive torque in all the rotations axis. The adjustability of the hanging point at the base, and a set of sliding masses, allow us to achieve a quite accurate superposition of rotation point and center of mass for a quasi-neutral equilibrium. The testbed is completed by a PC workstation, to generate and stream the desired angular rates of the wheels, and a motion capture system for attitude determination.

## 1. Introduction

Interest in small satellite missions has increased in the last decades and the recent technological developments related to the CubeSats standard have drastically reduced the cost of producing and flying a satellite mission. This has been possible because of their relatively short development time, low cost and usage of the latest technologies [1]. Moreover, small satellites are characterized by light mass and small dimensions, allowing multiple CubeSats to be launched at once, and consequently an overall decrease in the cost of launching. The increased availability and use of these technologies bring the attention to the problem of hardware in-the-loop emulation of the functional space environment for the validation of the spacecraft (S/C) subsystems. Notably, Attitude, Determination, and Control Subsystems (ADCS) are to be subjected beforehand to on-ground validation by performing experimental campaigns on dedicated experimental testbeds.

Numerous S/C maneuver simulators and testbeds have been developed in academic institutions, government facilities, and industry laboratories. As reported in [2], S/C simulators can be divided into two types, kinematics, and dynamics simulators. The difference between the two is on the use of actuators that are (dynamics) or are not (kinematics) representative of the actuators used in real flight. Of particular interests are S/C attitude testbeds specifically devoted to on-ground validation and software development of ADCS. Most of these testbeds are composed of a hollow rigid hemisphere floating on a spherical pedestal through which air flows. This configuration emulates the external torque-free environment that S/C experience during their missions [3]. The Microsatellite and Space Microsystems Lab at University of Bologna has developed a three degrees-of-freedom (DoF) testbed, aimed at nanosatellites’ ADCS hardware and algorithms’ verification [4]. The testbed is based on a custom designed table-top air bearing platform, an automatic balancing system, a Helmholtz cage, a Sun simulator, and a metrology vision system. Another air-bearing S/C simulator is the nanosatellite-scale spacecraft three-axis simulator (CubeTAS) developed at the Naval Postgraduate School [5], also consisting of a hollow hemispherical structure containing actuators, sensors, an on-board computer and a battery. Similarly, the Laboratory of Application and Innovation in Aerospace Science of the University of Brasília is developing an air-bearing tabletop testbed equipped with Helmholtz cage to reproduce nanosatellite attitude motion [6]. The Institute of Space Systems of German Aerospace Center has developed the Facility for Attitude Control Experiments (FACE), a three-axis disturbance-free attitude control experiment platform, consisting of a hemispherical air-bearing with a satellite component platform, a preliminary solar simulator and a magnetic field simulator [7]. The King Mongkut’s University of Technology North Bangkok in Thailand developed a low-cost HIL simulator for testing the flight software and hardware of the ADCS subsytem of a CubeSat satellite, named KNACKSAT (KMUTNB Academic Challenge of Knowledge SATellite) [8]. Moreover, Ref. [9] proposed a new plan for attitude determination and control testing, based on the integration of the ADCS hardware components and data transmitting between the different parts. The setup provided better efficiency and cost reduction in hardware resources, reducing the disturbances of different simulators and promoting the development of other sensors and actuators environments. A different approach is used in [10], in which a suspended low-cost test-bed for attitude control and estimation is presented. The testbed is developed at the The Texas A&M Land, Air, and Space Laboratory (LASR), and it utilizes a neutrally stable universal joint to allow for three degrees of freedom attitude motion. The idea is to build a low-cost testbed suitable for educational and research purposes, by integrating a motion capture system as sensors, MATLAB software [11], a Raspberry Pi as single-board computer and custom reaction wheels (RW).

In this paper, we present the Suspended Satellite Three-Axis Rotation Testbed (START). This is a satellite attitude simulator testbed for on-ground experimentation of attitude, determination and control methodologies, located in the Aerospace Robotics Testbed Laboratory (ARTLAB) at South Dakota State University. This setup aims to be a low-cost alternative to testbeds based on air-bearing couplings. START is mainly composed of a 3D printed base, a single-board computer, a set of actuators and an electric battery. The suspension system is based on three thin high tensile strength wires converging on a single wire, which is in turn connected to the base. This configuration allows a 3-DoF rotation range comparable to that of air bearing-based floating testbeds, and minimal resistive torque in all the rotations axis. In contrast to other S/C simulators, such as air-based floating testbeds, the wired suspension system drastically reduces the development costs, maintaining the ability to achieve at least a comparable rotation range around the roll and pitch axes. The absence of residual viscous forces from airflow on bearing, and the simplicity in the development and assembly of the presented testbed, because of the extensive use of commercial off-the-shelf (COTS) components, are remarkable advantages for educational and research purposes. To the best of our knowledge, the testbed described in [10] is the only suspended attitude testbed that shares some characteristics with the START testbed hereby presented. The main differences are in the design of the platform, the components configuration, the suspension system, the motion capture system, and in the choice of the RWs and their sizing.

The paper is organized as follows. Section 2 reports an overview of the different START’s subsystems, with a focus on the hardware components and integration of the satellite simulator. Section 3 describes the START’s software architecture. Section 4 presents the attitude determination and control subsystems, together with the results and discussion of the preliminary testing. Lastly, in Section 5 final remarks are provided to conclude the paper.

## 2. Hardware

In this section, we describe the main components and integration of the satellite simulator, focusing on the general hardware setup of the testbed, the development of the 3D printed base, the testbed balancing process, the characterization of the set of actuators adopted and the motion capture system.

### 2.1. Overview

**Figure 1:**
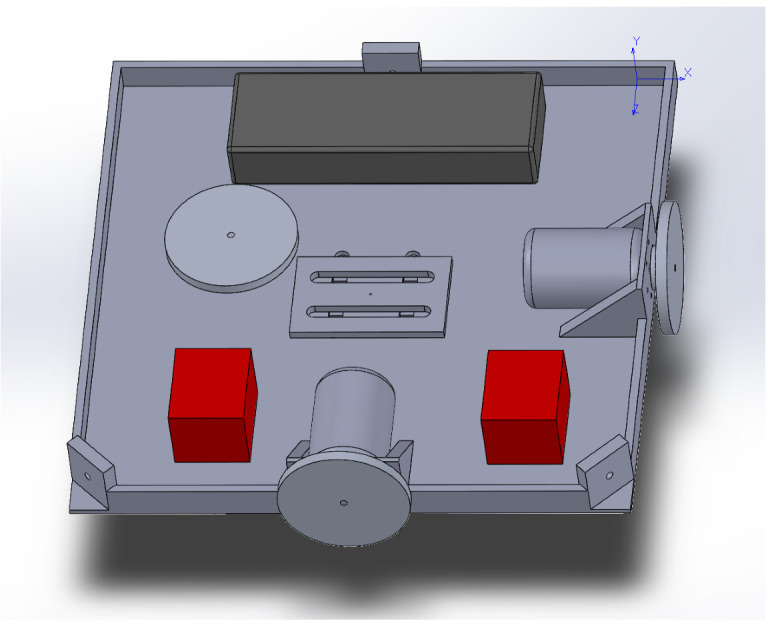
SOLIDWORKS CAD rendering of START.

**Figure 2:**
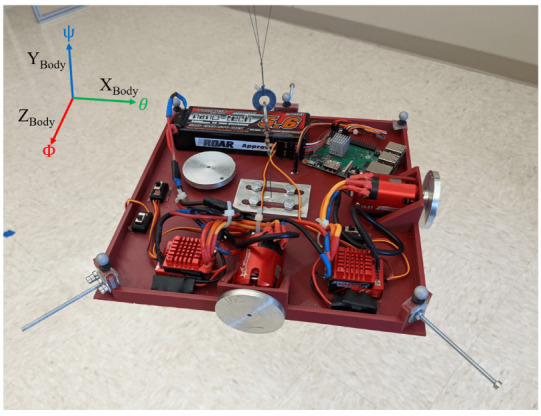
START and *Body-fixed* reference frame.

START is mainly composed of a 3D printed base, a single-board computer, a set of actuators, and an electric battery, as shown in Figure 1 and Figure 2.

The actuation package is composed of an assembly of three RWs. Each RW consists of a brushless direct current electric (BLDC) sensored motor, namely a TrackStar 13.5T motor and electronics speed controller (ESC) combo [12], and a custom manufactured aluminum alloy flywheel. The electric battery is a Turnigy nano-tech 5600mah 2S2P lithium-ion polymer rechargeable battery [13], and it is used to power the single-board computer and the RWs assembly. The single-board computer, a Raspberry Pi 3 Model B+ [14], is used to control the actuators, and it is the link between the MATLAB/Simulink software [11], the OptiTrack Motive Motion Caption System (MCS) [15], and the RWs. The testbed parameters and specificatons are listed in Table 1. Regarding the moment of inertia of START, these are determined through a SOLIDWORKS [16] CAD model comprehensive of all the major components, increased by a 5% margin to include unmodeled minor components, such as wires, switches and so forth. The total cost of the components of the satellite simulator platform assembly is about $500.

**Figure 3:**
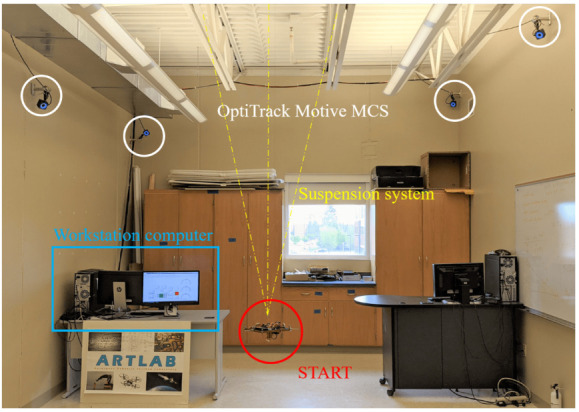
Picture of START in the Aerospace Robotics Testbed Laboratory (ARTLAB).

The base is suspended through three thin high tensile strength wires connected to the ceiling of ARTLAB, an experimental facility in the department of Mechanical Engineering at South Dakota State University (see Figure 3). Two reference frames are considered. The *Inertial* reference frame, determined during the calibration process of the OptiTrack Motive MCS, and the *Body-fixed* reference frame. In particular, the *Body-fixed* reference frame is centered in the center of mass of the platform, its axes are coincident to the body’s principal axis of inertia, and they follow the same conventions adopted by the *Inertial* reference frame. As shown in Figure 2, the $X_{\mathrm{Body}}$, $Y_{\mathrm{Body}}$, and $Z_{\mathrm{Body}}$ axes are associated to the pitch (θ), yaw (ψ), and roll (ϕ) rotations, respectively. The current testbed setup allows unlimited yaw rotation and a near $\pm 60{}^{\circ }$ rotation range around the roll and pitch axes.

### 2.2. Rotating Base

**Figure 4:**
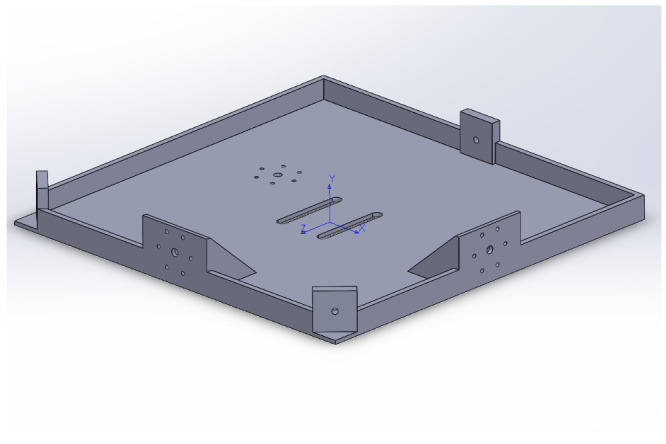
SOLIDWORKS CAD base model, axonometric view.

**Figure 5:**
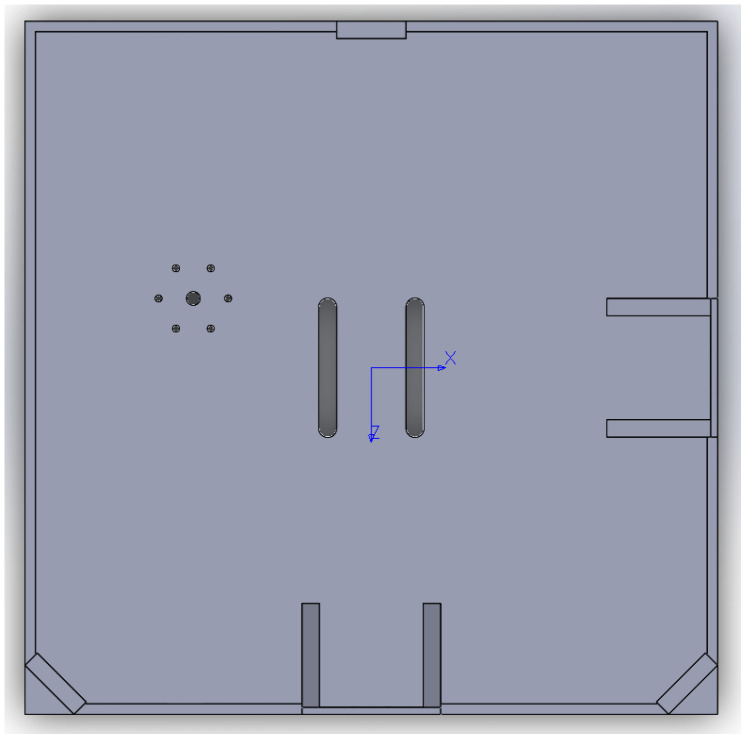
SOLIDWORKS CAD base model, top view.

The 3D printed platform is made of polylactic acid (PLA plastic), of dimensions 0.255 m × 0.255 m × 0.0025 m. In Figure 4 and Figure 5, the SOLIDWORKS CAD model rendeering is reported. As shown in Figure 2 and Figure 3, three thin high tensile strength fishing wires (max load 220 N) compose the suspension system. The connection between the testbed and the three wires is done by the use of a swivel, allowing the testbed to be easily removed from the suspension system, in case it necessitates of maintenance, of battery recharging, or for storing. Moreover, another set of high tensile strength wires connects the removable swivel to an adjustable hanging point, mounted on the 3D printed base. The adjustable hanging point is an aluminum top mount, as shown in Figure 2, that accommodates the wires passing through a hole. This hanging point can be adjusted on the $X_{\mathrm{Body}}$-$Z_{\mathrm{Body}}$ plane by displacing the mount. The goal is to have a perfect alignment, on the $Z_{\mathrm{Body}}$ axis, between the center of mass of the base with the hanging point which constitute the center of rotation. This setup guarantees that the testbed can perform three-axis maneuvers with minimal external resistive torque. In addition to the adjustable hanging point, three rods at the edges of the base, and one at the bottom, are used to accommodate additional masses in order to complete the balancing process of the testbed. A set of sliding masses (set of nuts and small magnets) are accurately placed to achieve a quite accurate superposition of the rotation point and the center of mass for a quasi-neutral equilibrium. In particular, the static balancing is obtained by carefully moving the sliding masses along their respective rods until the base is horizontally levelled, and the oscillations, voluntarily triggered by manually perturbing the levelled position, are characterized by a long period. This behavior ensures that the center of mass of the platform is slightly below the center of rotation. At the end of the balancing process, the total mass of START results in 1.675 kg.

### 2.3. Reaction Wheels

**Figure 6:**
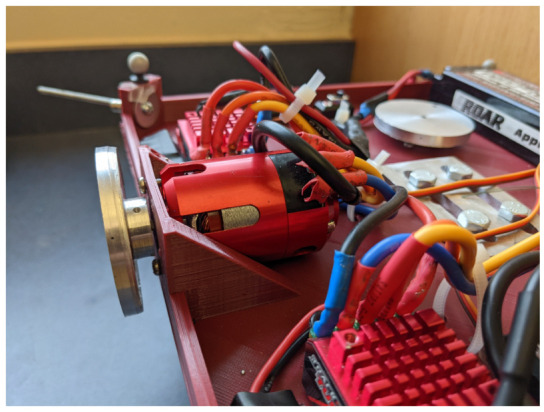
TrackStar 13.5T Brushless Motor and ESC Combo with custom manufactured flywheel.

**Figure 7:**
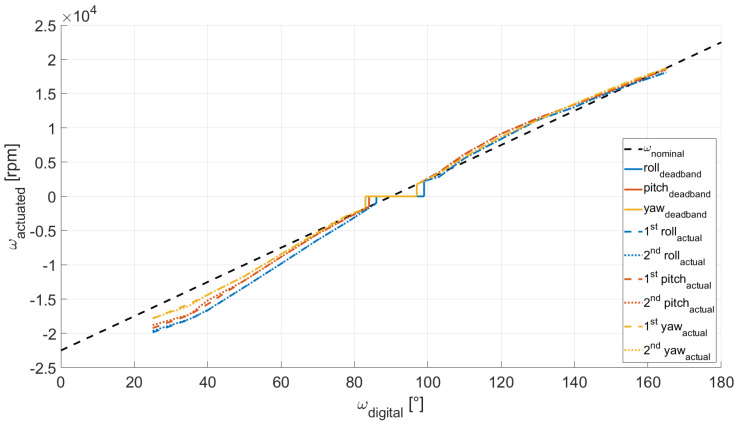
BLDC motors characteristic curves.

The actuation package is composed of a set of three RWs, each of them consisting in a sensored BLDC motor and a custom made flywheel (see Figure 6). The RWs are mounted parallel to the direction of the *Body-fixed* reference frame axes, as shown in Figure 2. The flywheels are made of aluminum alloy and have the following specifications: 0.05 m diameter, 0.005 m thickness, 0.035 kg weight and 0.00001739 kg m^2^ moment of inertia about its axis of symmetry. The wheels are secured to the motor shaft via a set screw. They can be controlled through the MATLAB/Simulink support package for Raspberry Pi hardware, and, in particular, through the servo motors function, once a connection to the Raspberry Pi hardware is established. The BLDC motors can turn in either direction and they are controlled via a digital input which correspond to the rotation angle of a fictitious equivalent servo motor. Such a command is mapped into the pulse width modulation duty cycle duration. In our case, 1 ms pulse-width maps to a servo motor angle of $0{}^{\circ }$, corresponding to a clockwise rotation equal to −22,496 rpm, whereas 2 ms pulse-width maps to a servo angle of $180{}^{\circ }$, corresponding to a counter-clockwise rotation equal to 22,496 rpm. It must be noted that each of the three motors is characterized by a dead band, namely the wheel will not rotate for small input commands in one direction and the other. Moreover, the motor angular rate suffers a quantization error due to the discretization of the digital input. To properly characterize the correspondence between the requested input and the actuated angular rate, we measured the rotation speed of each motor by using a tachometer, while maintaining a battery level in the range 7.90–8.15 V, to mitigate the effects related to drop of the battery level. In Figure 7, the motor characteristic curve for the three BLDC motors is reported. The rotation of every wheels is described in terms of angular rate, divided into actuated and digital angular rate, $\omega_{actuated}$ and $\omega_{digital}$ respectively. We refer to the digital angular rate as the servo angle set in order to obtain a specific rotation, being able in this way to control via software the actuators. The actuated angular rate is, instead, the conversion of the servo angle into revolutions per minute (rpm). As depicted in Figure 7, two sets of measurements per each motor are carried out, and the measured angular rate results similar to the nominal expected behavior. Notably, we can observe only minor deviations, from one experiment to another, of actual angular rates correspondent to the same digital input.

### 2.4. Motion Capture System

**Figure 8:**
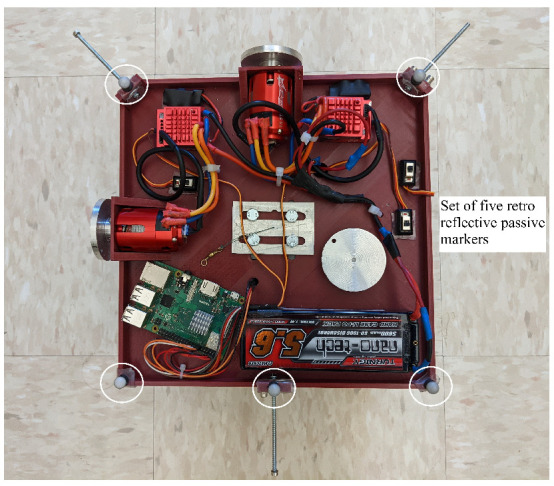
Set of five retro reflective passive markers attached to the START.

**Figure 9:**
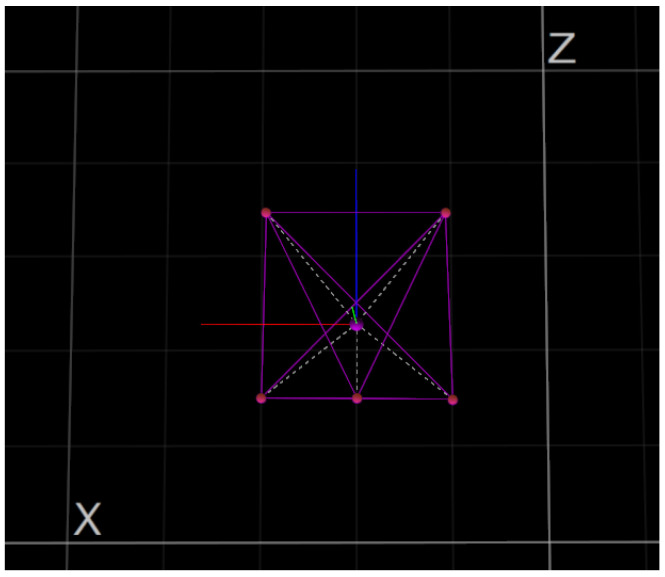
OptiTrack Motive MCS—rendering.

The MCS is based on eight OptiTrack Prime 13 cameras (see Figure 3) [17] with the following specifications: a resolution of 1.3 megapixels, a frame rate of 240 frames per second, and a field of view of $42.56{}^{\circ }$. The cameras allow the tracking of real-time position and attitude of a rigid body using a set of retro reflective passive markers. To create a rigid body, at least three retro reflective passive markers are necessary. As shown in Figure 8 and Figure 9, five markers are attached to the START platform which constitute the rigid body. The software is capable to stream the position and attitude data retrieved by the cameras in real-time. A Level-2 MATLAB S-Function [18] is used to gather the captured data from OptiTrack Motive MCS and to stream them to Simulink at a 100 Hz stream rate.

## 3. Software

In this section, an overview of the START’s software implemented is carried out, focusing on the software architecture and on the Simulink models.

### 3.1. Architecture

**Figure 10:**
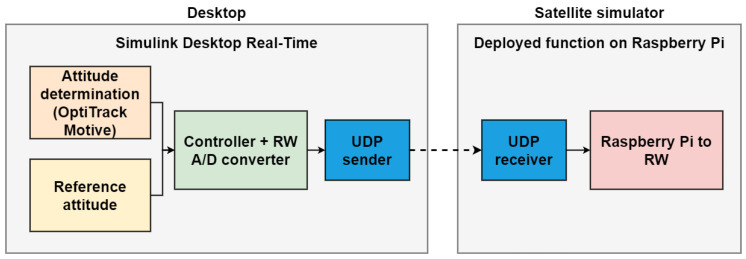
Software architecture block scheme.

Two Simulink models, as depicted in Figure 10, have been developed. The first one is a function that is deployed on the Raspberry Pi hardware, to enable the communication between the workstation computer and the actuators. The second model runs on the workstation computer using the Simulink Desktop Real-Time feature [19], allowing the streaming of the control inputs—namely the RWs angular rates—retrieved by the attitude determination and control subsystems to the deployed model. In addition, it is possible to have real-time knowledge of the data streamed to the model, and of the resulting START attitude maneuver. The streaming of the control inputs from this model to the deployed one is via the user data protocol (UDP).

### 3.2. Simulink Models

**Figure 11:**
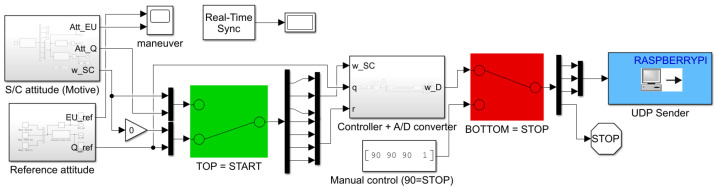
Simulink Desktop Real-Time model running on the PC workstation.

**Figure 12:**
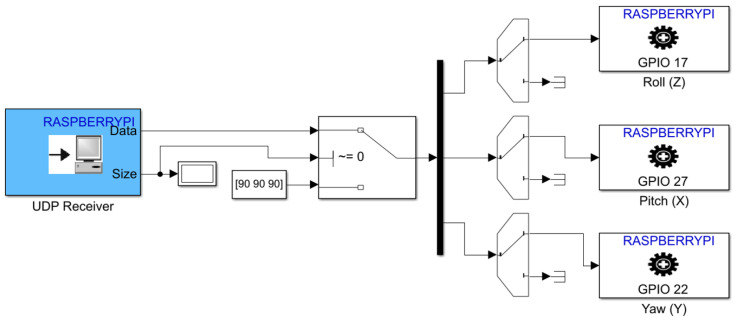
Simulink deployed function on the Raspberry Pi.

Figure 11 and Figure 12 show in detail the two Simulink models developed to control START. The first one is running on the workstation computer using Simulink Desktop Real-Time feature, whereas the second one is deployed on the Raspberry Pi hardware. As shown in Figure 11, the first model is characterized by three main subsystem blocks, namely the S/C attitude determination, the reference attitude, and the attitude control subsystem blocks. Through the use of a manual switch, it is possible to begin the attitude maneuver after START is manually hold steady by an operator, and the RW are initialized. Similarly, a second manual switch allows us to stop the attitude maneuver by setting a value of 90 for each digital RW angular rate. Finally, a UDP Send block is utilized to stream the control inputs, in terms of digital RW angular rates, through wireless protocols to the Simulink function deployed on the Raspberry Pi hardware. In this way, a communication between the workstation computer and the actuators is enabled. Regarding the Simulink function deployed on the Raspberry Pi, as shown in Figure 12, a UDP Receive block is used to receive the UDP packets from the first model. The packets of digital RW angular rates are then given as inputs to each RW by using the Standard Servo Write block, which allows us to control the shaft angle of a motor attached to a pulse width modulation (PWM) output pin on the Raspbery Pi hardware board.

## 4. ADCS and Preliminary Testing

In this section, we describe the attitude determination and control subsystems, and the preliminary testing and validation of START involving two attitude maneuvers, using the hardware and software setup previously described.

For our initial testbed experimentation, we executed a rest-to-rest maneuver to reorient the platform from an initial attitude to a final commanded attitude with null final angular rates. The attitude control strategy is a quaternion feedback Proportional-Derivative (PD) controller. The PD gains are tuned to achieve favorable performance in terms of settling time and final accuracy. Notably, the quaternion feedback PD controller combines the proportional contribution on the quaternion error between the *Body* and the commanded attitude, the derivative contribution on the angular velocity error between the *Body* and the commanded angular velocity, and a nonlinear body-rate feedback term that counteracts the gyroscopic coupling torque [20].

(1) $$u=K_{p}\frac{\partial H(q_{e_{0}})}{\partial q_{e_{0}}}Jq_{e}-K_{d}J\omega_{e}+\omega_{Body}\times J\omega_{Body}$$

(2) $$H\left(q_{e_{0}}\right)=1-\mathrm{sign}\left(q_{e_{0}}\right)q_{e_{0}}$$

(3) $$J=\left[\begin{array}{ccc} J_{x} & 0 & 0\\ 0 & J_{y} & 0\\ 0 & 0 & J_{z} \end{array}\right],\;K_{p}=\left[\begin{array}{ccc} k_{p_{\phi }} & 0 & 0\\ 0 & k_{p_{\theta }} & 0\\ 0 & 0 & k_{p_{\psi }} \end{array}\right],\;K_{d}=\left[\begin{array}{ccc} k_{d_{\phi }} & 0 & 0\\ 0 & k_{d_{\theta }} & 0\\ 0 & 0 & k_{d_{\psi }} \end{array}\right],$$

with $H(q_{e_{0}})$ a function that satisfies the Lyapunov Stability theorem, J the inertia matrix, $q_{e}$ the quaternions error vector between the *Body* frame and the commanded attitude, $\omega_{e}$ the angular velocity vector between the *Body* and the commanded angular velocity, and $K_{p}$, and $K_{d}$ the proportional, and the derivative gains matrices, respectively. The quaternion error ${\hat{q}}_{e}={\left[q_{e_{0}},q_{e}^{T}\right]}^{T}$, which is characterized by a scalar part $q_{e1}$ and a vector part $q_{e}={\left[q_{e_{1}},q_{e_{2}},q_{e_{3}}\right]}^{T}$, is defined as:

(4) $${\hat{q}}_{e}={\hat{q}}_{c}^{-1}⊗{\hat{q}}_{Body},$$

where ⊗ denotes the quaternion multiplication, ${\hat{q}}_{c}$ is the commanded attitude in quaternion form, and ${\hat{q}}_{Body}$ the actual *Body* attitude. The *Body* angular velocity is retrieved by inverting the quaternions attitude kinematic equations, as follows [20]

(5) $${\left[0,\omega_{e}\right]}^{T}=2\frac{d{\hat{q}}_{Body}}{dt}{\bar{q}}_{Body},$$

where ${\bar{q}}_{Body}$ is the the conjugate (or inverse, since unit quaternion) of the actual *Body* attitude ${\hat{q}}_{Body}$, and $\frac{d{\hat{q}}_{Body}}{dt}$ its numerical derivative.

Lastly, to retrieve the RW actuated and digital angular rate, $\omega_{actuated}$ and $\omega_{digital}$, partitioned on the three axes and commanded to the BLDC motors, we use the following relations [21]:

(6) $$\omega_{actuated}=\frac{h_{RW}}{I_{RW}}$$

(7) $${\dot{h}}_{RW}=A^{-1}\left(Ah_{RW}\times \omega_{Body}-u\right)$$

(8) $$A=\left[\begin{array}{ccc} 1 & 0 & 0\\ 0 & 1 & 0\\ 0 & 0 & 1 \end{array}\right],$$

where $I_{RW}$ is the RW moment of inertia, $h_{RW}$ is the RW’ angular momentum around its spin axis, and A is the RW orientation matrix, with as many columns as the number of RWs, and each column represents the direction of the axis of rotation of the wheel.

Regarding the attitude determination subsystem, in our application, we are interested in knowing the attitude and the angular velocity of the START at every sample time interval. The OptiTrack Motive MCS is able to track only the real-time position and attitude of a rigid body. For this reason, we numerically retrieve the rigid body’s angular velocity from the real-time attitude data, expressed in quaternions, by inverting the quaternions attitude kinematic equations, as shown in Equation (5). A low-pass filter is also implemented to mitigate the high frequencies from the angular velocities signal. As result, we attain the START attitude and angular velocity, which are the inputs of the attitude control subsystem.

The preliminary testing and validation of START involve two attitude maneuvers that are, respectively, a rest-to-rest yaw maneuver and a rest-to-rest two-axis pointing maneuver. Table 2 reports the initial and final conditions, in terms of attitude and angular velocity, for the two attitude maneuvers.

It must be noted that the initial conditions, in terms of attitude and angular velocity, are near zero but are not perfectly null since the base is manually held steady by an operator at the beginning of the maneuver inducing a residual motion as the platform is released. Table 3 reports the quaternion feedback PD controller’s gains for each attitude channel used in the two cases. In this paper, interest is on an initial testing of the START capabilities, to understand if possible adjustments to the hardware or the balancing process are necessary to enhance its performances. For this reason, we are still exploring different gains and settings.

The experiments are performed using the Raspberry Pi together with MATLAB-Simulink software, and OptiTrack Motive MCS running on a workstation computer with the following specs: Intel(R) Core(TM) i7-6700 CPU 3.40 GHz processor, 16.0 GB of RAM, running a 64-bit Windows 10 operating system.

### 4.1. Case 1: Rest-to-Rest Pointing Yaw Maneuver

**Figure 13:**
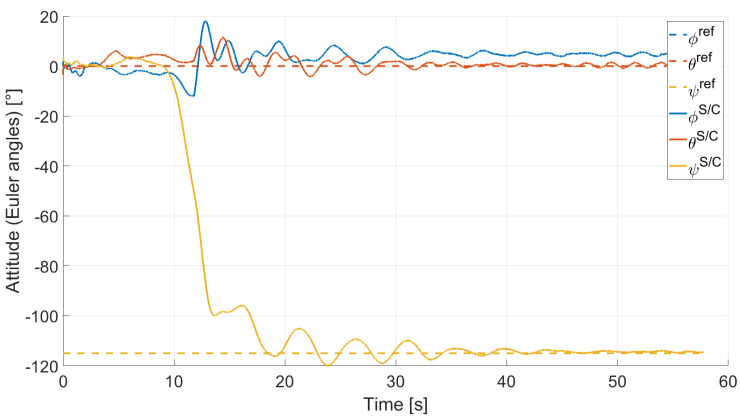
Case 1: attitude as Euler angles.

**Figure 14:**
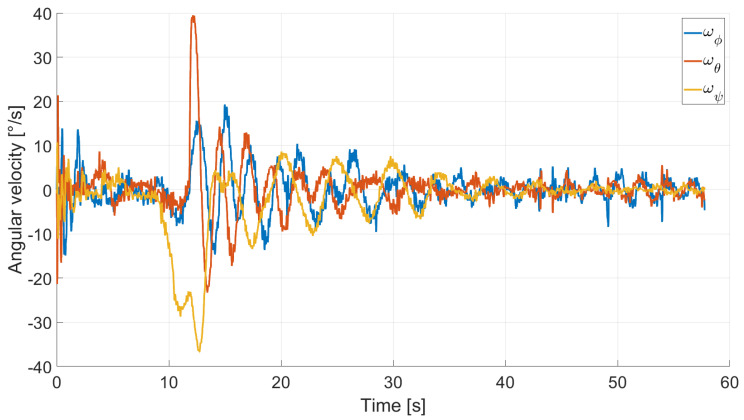
Case 1: angular velocity.

**Figure 15:**
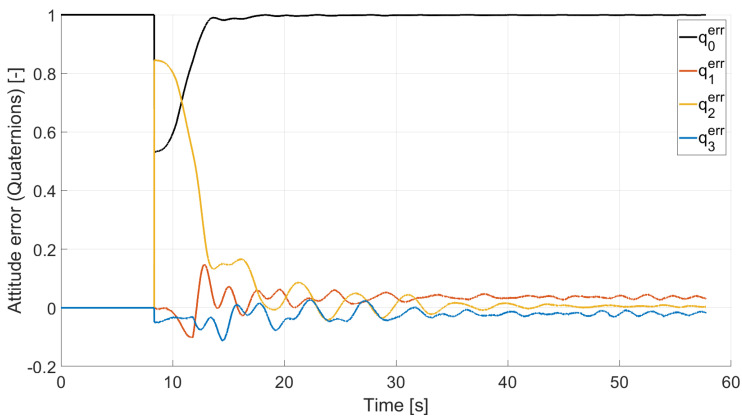
Case 1: quaternion error.

**Figure 16:**
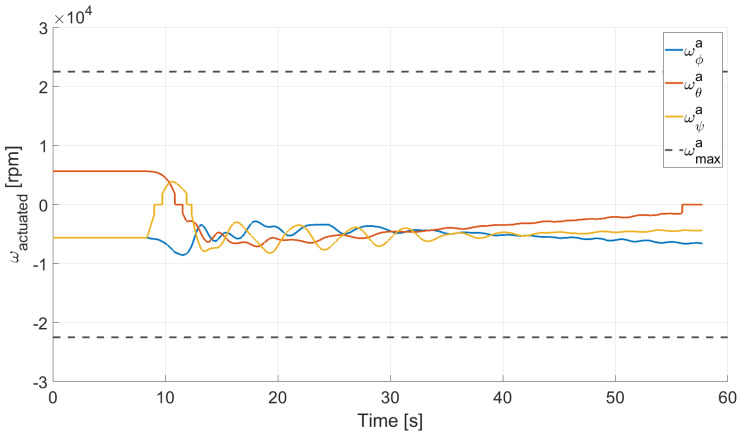
Case 1: RW actuated angular rate.

In Case 1, we tested a rest-to-rest pointing yaw maneuver. To reproduce the initial conditions as close as possible to the desired ones, the RWs are initialized at a constant angular rate while the testbed is manually held still. After approximately 8 s, a commanded switch initiate the attitude maneuver by enabling RW angular rate changes according to the control law. Figure 13, Figure 14, Figure 15 and Figure 16 report the results for this maneuver. In particular, Figure 13 and Figure 14 depict the attitude (as Euler angles) and the angular velocity of the testbed, Figure 15 the attitude error of the testbed with respect of the reference attitude trajectory (as quaternion error), and Figure 16 the RWs actuated angular rate. The rest-to-rest yaw maneuver takes approximately 50 s to complete. Pitch (θ) and yaw (ψ) axes reach the reference attitude achieving a pointing accuracy of about $\pm 1.5{}^{\circ }$ and $\pm 1{}^{\circ }$, respectively. On the contrary, the roll (ϕ) axis is a bit off from the commanded attitude. Regarding the angular velocity, indeed, it is possible to see how oscillations around the reference value are present. This can be solved by improving the residual testbed balancing and by tuning further the PD gains.

### 4.2. Case 2: Rest-to-Rest Pointing Two-Axis Maneuver

**Figure 17:**
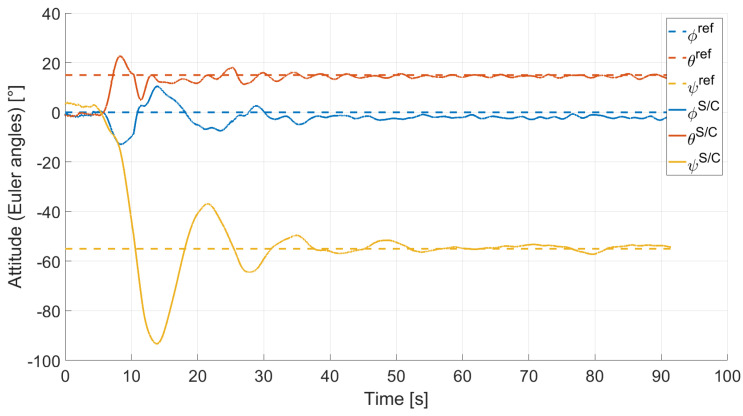
Case 2: attitude as Euler angles.

**Figure 18:**
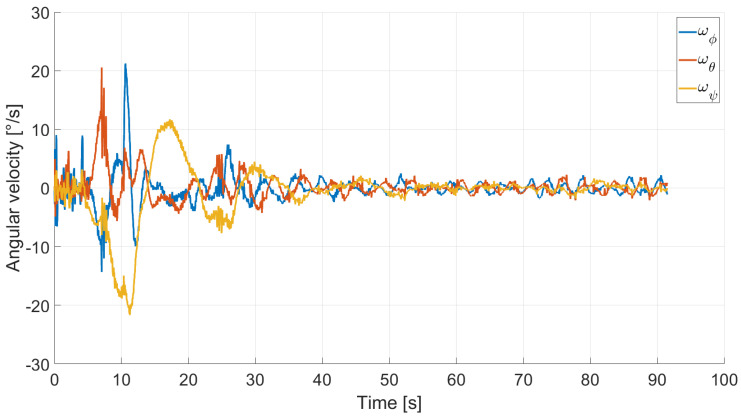
Case 2: angular velocity.

**Figure 19:**
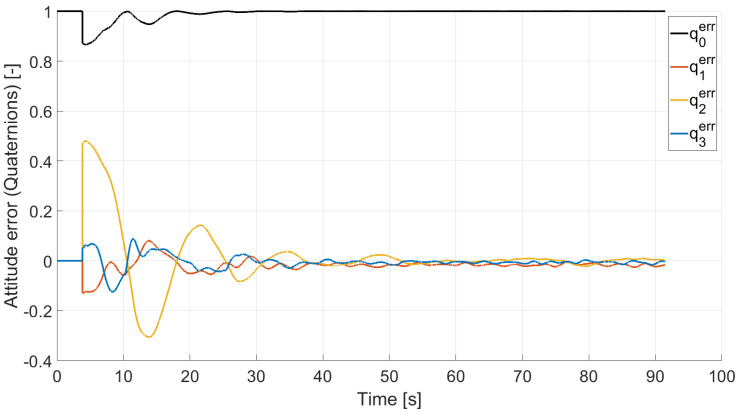
Case 2: quaternion error.

**Figure 20:**
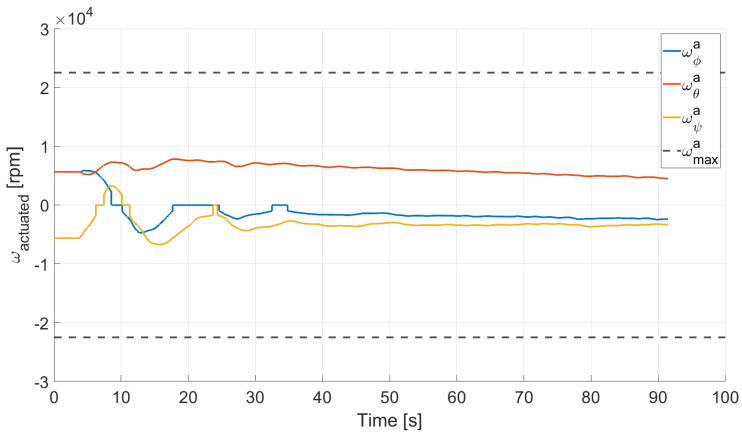
Case 2: RW actuated angular rate.

In Case 2, we tested a rest-to-rest pointing two-axis maneuver. To reproduce the initial conditions as close as possible to the desired ones, the RWs are initializated at a constant angular rate, and consequently produces torque, while the testbed is kept still. After approximately 4 s, a commanded switch toggles the attitude maneuver. Figure 17, Figure 18, Figure 19 and Figure 20 report the results for this maneuver. In particular, Figure 17 and Figure 18 depict the attitude (as Euler angles) and the angular velocity of the testbed, Figure 19 the attitude error of the testbed with respect of the reference attitude trajectory (as quaternion error), and Figure 20 the RWs actuated angular rate. The rest-to-rest pointing maneuver takes approximately 80 s to complete. As results, the pitch (θ) and yaw (ψ) axes reach the reference attitude achieving a pointing accuracy of about $\pm 1.5{}^{\circ }$. On the contrary, the roll (ϕ) axis is a bit off from the commanded attitude. Regarding the angular velocity, indeed, it is possible to see how oscillations around the reference value are present. As stated in the previous case, this can be solved by improving the testbed residual balancing, by further tuning the PD gains and by refining the motor settings to be more suitable for a RW-like behaviour.

## 5. Conclusions

In this paper, we presented the Suspended Satellite Three-Axis Rotation Testbed (START), which is a novel low-cost satellite three-axis attitude simulator testbed. This platform is mainly composed of a 3D printed base, a single-board computer, a set of actuators, and an electric battery. The testbed is completed by a motion capture system and a PC workstation. This setup has been initially tested considering two attitude maneuvers, a rest-to-rest pointing yaw maneuver, and a rest-to-rest pointing two-axis maneuver. From the analysis of the results, we can conclude that the testbed subsystems and software were successfully implemented, and that the quaternion feedback PD controller was able to perform the rest-to-rest pointing maneuvers. In terms of final accuracy, the maneuvers were able to achieve reasonable results, considering the low-cost budget allocated to build and develop the testbed. As discussed in the results section, to improve the results, we could consider refining the testbed balancing, fine-tuning the controller’s gains, and refining the motors setting to have them perform closely to a reaction wheel-like behavior. Once these points are addressed, further potential research using the developed testbed could involve the ground testing of advanced attitude determination and control algorithms, the use different actuators such as cold gas thrusters, a new testbed platform similar to the CubeSat standard shape, and the implementation of on-board attitude estimation sensors.

## Figures and Tables

**Table 1:** START specifications.

Data	Value	Data	Value
START mass	1.675 kg	Max RW DC voltage	7.4 V
Moment inertia $J_{x}$	$6.5\;\times \;10^{-3}\;$ $\mathrm{kg}\;m^{2}$	Max RW current	36 A
Moment inertia $J_{y}$	$13.6\;\times \;10^{-3}\;$ $\mathrm{kg}\;m^{2}$	Max RW torque	0.1082 Nm
Moment inertia $J_{z}$	$7.8\;\times \;10^{-3}\;$ $\mathrm{kg}\;m^{2}$	Max RW momentum storage	0.0576 Nms
Moment inertia $J_{RW}$	$2.45\;\times \;10^{-5}\;$ $\mathrm{kg}\;m^{2}$	Max RW $\omega_{actuated}$	22,496 rpm

**Table 2:** Attitude maneuvers.

	Initial Conditions		Final Conditions	
Data	ϕ,θ,ψ [${}^{\circ }$]	$\omega_{\mathrm{Body}}$ [${}^{\circ }/s$]	ϕ,θ,ψ [${}^{\circ }$]	$\omega_{\mathrm{Body}}$ [${}^{\circ }/s$]
Case 1	[0,0,0]	[0,0,0]	[0,0,−115]	[0,0,0]
Case 2	[0,0,0]	[0,0,0]	[0,15,−55]	[0,0,0]

**Table 3:** Quaternion feedback PD controller’s gains.

Roll		Pitch		Yaw	
$k_{p_{\phi }}$	$k_{d_{\phi }}$	$k_{p_{\theta }}$	$k_{d_{\theta }}$	$k_{p_{\psi }}$	$k_{d_{\psi }}$
1.4	2.38	1.4	2.38	1.4	2.73

## References

[1] Bouwmeester J., Guo J. (2010). Survey of worldwide pico- and nanosatellite missions, distributions and subsystem technology. Acta Astronaut..

[2] Wilde M., Clark C., Romano M. (2019). Historical survey of kinematic and dynamic spacecraft simulators for laboratory experimentation of on-orbit proximity maneuvers. Prog. Aerosp. Sci..

[3] Schwartz J.L., Peck M.A., Hall C.D. (2003). Historical Review of Air-Bearing Spacecraft Simulators. J. Guid. Control. Dyn..

[4] Modenini D., Bahu A., Curzi G., Togni A. (2020). A Dynamic Testbed for Nanosatellites Attitude Verification. Aerospace.

[5] Chesi S., Perez O., Romano M. (2015). A Dynamic Hardware-in-the-loop Three-Axis Simulator of Nanosatellite Dimensions. J. Small Spacecr..

[6] da Silva R.C., Guimarães F.C., de Loiola J.V.L., Borges R.A., Battistini S., Cappelletti C. (2019). Tabletop Testbed for Attitude Determination and Control of Nanosatellites. J. Aerosp. Eng..

[7] Kato T., Heidecker A., Dumke M., Theil S. (2014). Three-Axis Disturbance-Free Attitude Control Experiment Platform: FACE. Trans. Jpn. Soc. Aeronaut. Space Sci. Aerosp. Technol. Jpn..

[8] Tapsawat W., Sangpet T., Kuntanapreeda S. (2018). Development of a hardware-in-loop attitude control simulator for a CubeSat satellite. IOP Conf. Ser. Mater. Sci. Eng..

[9] Tavakoli A., Faghihinia A., Kalhor A. (2017). An Innovative test bed for verification of attitude control system. IEEE Aerosp. Electron. Syst. Mag..

[10] Hurtado J., Goecks V.G., Probe A. Low-Cost Satellite Attitude Hardware Test Bed. Proceedings of the ASEE Annual Conference and Exposition.

[11] (2020). MATLAB.

[12] (2020). TrackStar ROAR Approved 1/10th Stock Class Brushless ESC and Motor Combo (13.5T).

[13] (2020). Turnigy Nano-Tech 5600mah 2S2P 50 100C Hardcase Lipo Pack (ROAR APPROVED).

[14] (2020). Raspberry Pi 3 Model B+.

[15] (2020). OptiTrack Motive Motion Capture Software.

[16] (2020). SOLIDWORKS.

[17] (2020). Prime 13.

[18] Cherfan R. (2021). MotiveExample. MATLAB Central File Exchange.

[19] (2020). Simulink Desktop Real-Time.

[20] Wie B. (2008). Space Vehicle Dynamics and Control.

[21] Markley F.L., Crassidis J.L. (2014). Fundamentals of Spacecraft Attitude Determination and Control.

